# The Spectral-Domain Optical Coherence Tomography Findings Associated with the Morphological and Electrophysiological Changes in a Rat Model of Retinal Degeneration, Rhodopsin S334ter-4 Rats

**DOI:** 10.1155/2018/5174986

**Published:** 2018-11-15

**Authors:** Kodai Yamauchi, Reiko Tanabu, Natsuki Monai, Takayuki Gonome, Yuting Xie, Shizuka Takahashi, Sei-ichi Ishiguro, Mitsuru Nakazawa

**Affiliations:** ^1^Department of Ophthalmology, Hirosaki University Graduate School of Medicine, Hirosaki, Japan; ^2^Department of Ophthalmology, Tohoku University Graduate School of Medicine, Sendai, Japan

## Abstract

**Purpose:**

To characterize the spectral-domain optical coherence tomography (SD-OCT) findings of the rhodopsin S334ter transgenic rats (line 4) in relation to the morphologic and electroretinographic features.

**Materials and Methods:**

Rhodopsin S334ter transgenic rats (line 4) were employed as a model of retinal degeneration. The Sprague-Dawley (SD) rats were used as a wild-type control. SD-OCT (Micron IV®; Phoenix Research Labs, Pleasanton, CA, USA) was performed on the S334ter rats (line 4) from postnatal days (P) 13−110. The longitudinal changes of the SD-OCT images were analyzed both qualitatively and quantitatively in comparison to those of SD rats. The SD-OCT images were also compared to the histological and electron microscopic findings from examination performed on P 22, 36, and 61. Full field combined rod and cone electroretinography (ERG) was performed and the relationship between the thickness of the retinal sublayers and the amplitudes of the a- and b-waves was further analyzed.

**Results:**

The photoreceptor inner and outer segment layer became diffusely hyperreflective in the SD-OCT images of the S334ter rats; these findings were not observed in the SD rats. This hyperreflective change corresponded to the degenerated inner and outer segments and the accumulation of the extracellular vesicles in the interphotoreceptor matrix. Quantitatively, the retinal outer sublayer and the photoreceptor sublayer in the S334ter rats became progressively thinner in comparison to those in the SD rats; the difference was statistically significant. The amplitudes of both the a- and b-waves on ERG were severely deteriorated in the S334ter rats.

**Discussion:**

The SD-OCT images in the S334ter rats noninvasively provided information regarding the pathological changes in the photoreceptors and the longitudinal changes of both qualitative and quantitative changes during retinal degeneration in the S334ter rats (line 4). The pathological features of the photoreceptor inner and outer segments can be detected on SD-OCT as diffuse hyperreflective changes in the photoreceptor layer.

## 1. Introduction

Retinitis pigmentosa (RP) is the most common hereditary condition to cause retinal degeneration; it occurs in approximately 1 in 4,000 people worldwide [1]. RP is characterized by the primary progressive degeneration of the rod photoreceptors and the subsequent consecutive decline of the cone photoreceptors. Although the phenotypic features are heterogeneous, night blindness and photophobia are the typical initial symptoms and the progressive constriction of the visual field and decreased visual acuity are eventually follow.

The molecular genetic background of RP is heterogeneous. To date, more than 80 causative genes (RetNet: Disease Table: https://sph.uth.edu/retnet/disease.htm) have been identified for RP. Among these, the rhodopsin gene is the most common causative gene of the autosomal-dominant form of RP (ADRP). Mutations in the rhodopsin gene are detected in 25-30% of patients with ADRP [1, 2]. Even in patients with ADRP associated with mutations in the rhodopsin gene, the phenotypic features are variable; they roughly depend on the location of the mutation in the gene. Mutations are traditionally classified by the intradiscal, transmembrane, and cytoplasmic domains of the rhodopsin molecule [3]. Of these, mutations in the cytoplasmic domain (Class 1) are associated with more severe phenotypes in comparison to the other two domains [3, 4]. The Class 1 group consists of mutations causing defects in the C-terminal-end portion of the rhodopsin molecule, which possesses trafficking and quenching signals. Thus, the mechanisms of photoreceptor cell death account for (1) mislocalization of the rhodopsin molecule to the outer segment and (2) the constitutive activation of phototransduction [5, 6].

In the clinical field of ophthalmology, the recent development of spectral-domain optical coherence tomography (SD-OCT), a noninvasive imaging modality, has provided information of the fine structure of the retina. We previously reported the characteristic SD-OCT findings of retinal degeneration in Royal College of Surgeon's (RCS) rats with* mertk* gene mutations [7] and the rhodopsin P23H (line 2) transgenic rats [8]. In addition, the SD-OCT findings of the retinal degeneration that occurs in rhodopsin knockout mice [9], rats with rhodopsin P23H (line 1) mutation (Class 2) [10, 11], rd10 mice [12], arrestin knockout mice [13], and RCS rats [14] were reported previously. Because the mechanisms of cell death are highly heterogeneous and depend on the causative mutations [15], it is important to investigate the characteristics of photoreceptor degeneration associated with each causative gene mutation. We hypothesized that the SD-OCT findings of the retinal degeneration caused by different gene mutations may be different. To the best of our knowledge, there have been no reports on the SD-OCT characteristics of retinal degeneration caused by the rhodopsin Class 1 mutations. Although Thomas et al. reported the time domain OCT (TD-OCT) findings of transgenic S334ter rats (line 3) [16], the quality of the TD-OCT images was not enough to provide fine structure of the degenerated retina. In the present study, we attempted to characterize the SD-OCT findings of retinal degeneration in transgenic rhodopsin S334ter rats (line 4) [17], a typical Class 1 mutant, to seek any particular characteristics in the OCT findings in relation to the morphological and electrophysiological features.

## 2. Materials and Methods

### 2.1. Experimental Animals

The experimental procedures performed in this study conformed to the Association for Research in Vision and Ophthalmology (ARVO) Statement for the Use of Animals in Ophthalmic and Vision Research and were approved by the institutional Committee of Ethics for animal experiments (Approval Number: M11026).

The homozygous rhodopsin S334ter transgenic rats (line 4) were generously provided by Dr. Mathew M LaVail of the University of California. Sprague-Dawley (SD) rats were used as wild type (wt) control rats and were purchased from Clea Japan (Tokyo, Japan). The rats were maintained at the Hirosaki University Graduate School of Medicine Animal Care Service facility under a cycle of 12 h of light (50 lx illumination) and 12 h of darkness (<10 lx environmental illumination) in an air-conditioning atmosphere. Care was taken not to cause light-induced photoreceptor damage. The animals had* ad libitum *access to food and water.

### 2.2. SD-OCT Examinations

SD-OCT was performed according to previously reported methods [8]. In brief, SD-OCT was carried out at 8 time points from postnatal day (P)13 to P110 for S334ter rats and at 6 points from P19 until P134 for SD rats. Three or four rats (six to eight eyes) were examined at each time point. Number of rats for each measurement is shown in Table 1. Rats were anesthetized with an intraperitoneal injection of a mixture of medetomidine hydrochloride (0.315mg/kg), midazolam (2.0mg/kg), and butorphanol tartrate (2.5mg/kg) after the induction of analgesia by the inhalation of 80% carbon dioxide and 20% oxygen. Using a Micron® IV (Phoenix Research Labs, Pleasanton, CA, USA), SD-OCT images were obtained from a position set horizontally at 1 disc diameter superior to the optic disc under simultaneous ocular fundus monitoring. The pupils were dilated with the instillation of eye drops containing a mixture of 0.5% tropicamide and 0.5% phenylephrine hydrochloride. The corneal surface was protected using a 1.5% hydroxyethylcellulose solution. Fifty images were averaged to eliminate the projection artifacts. The acquired SD-OCT images were quantitatively analyzed using the InSight® software program (Phoenix Research Labs) after segmentation of each sublayer was manually performed by the researchers (KY, RT, NM, and MN). During all experimental procedures, the physical condition of the rats was frequently monitored by inspection and gentle palpation by the researchers.

### 2.3. The Analysis of the Retinal Layer Thickness (Segmentation)

**Figure 1:**
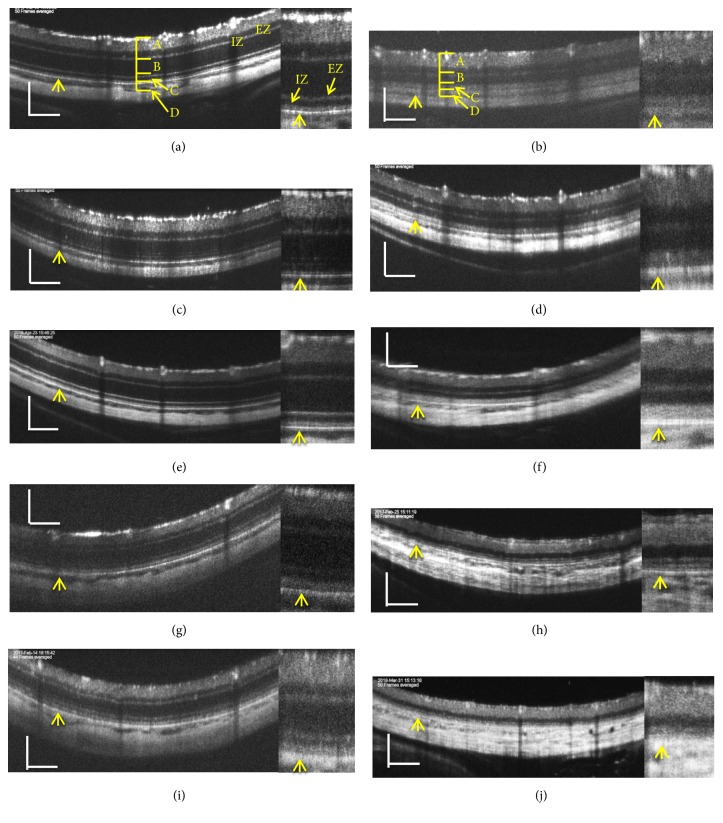
Representative pictures of spectral domain optical coherence tomography (SD-OCT) images of SD rats (a, c, e, g, and i) and S334ter rats (b, d, f, h, and j). (a) An SD rat on P19. (b) An S334ter rat on P19. (c) An SD rat on P22. (d) An S334ter rat on P21. (e) An SD rat on P33. (f) An S334ter rat on P34. (g) An SD rat on P82. (h) An S334ter rat on P87. (i) An SD rat on P134. (j) An S334ter rat on P110. The right side panel of each picture is a magnified image. Abbreviations: A, retinal inner layer; B, retinal outer layer; C, photoreceptor rod and cone layer; D, combined retinal pigment epithelium (RPE) and choroid layers; EZ, inner segment ellipsoid zone; IZ, interdigitation zone. Arrows indicate the RPE layer. Bar indicates 100 *μ*m.

We measured the thickness of the inner (A), the outer (B), and the photoreceptor (C) sublayers of the neural retina and the combined retinal pigment epithelium (RPE) and choroid layers (D, Figure 1(a), S 1). The definition of each layer was in line with previous reports [7, 8]. In brief, the inner layer A consisted of the retinal nerve fiber layer (NFL), the ganglion cell layer (GCL), the inner plexiform layer (IPL), and the inner nuclear layer (INL); the outer layer B includes the outer plexiform layer (OPL) and outer nuclear layer (ONL); and the photoreceptor rod and cone layer C consists of the photoreceptor inner segment (IS) and outer segment (OS) layers (Supplementary image 1).

As described in the previous section, the borderlines between each retinal sublayer were manually identified and segmented by the researchers. The average distance (*μ*m) between each borderline was then calculated using the raw data summarized from the InSight® software program. We used the average value of the data obtained from both eyes of the same animal and counted it as one observation. The overall average retinal layer thickness was presented as the mean ± standard error. The number of rats used in each measurement is described in Table 1 and Figure 4.

### 2.4. Histological Examination

Histological examinations were performed using eyes enucleated from S334ter rats on P22, P36, and P61 and SD rats on P69. Immediately after euthanasia by inhalation of 100% carbon dioxide, the eyes were excised under a microscope. To prevent the possibility of artificial retinal detachment occurring during further processing, an aliquot of 2% glutaraldehyde and 2% paraformaldehyde solution at pH 7.4 was injected into the anterior chamber through the corneal limbus. After fixation in the same solution for 2 h at room temperature, the eyeballs were refixed in 4% paraformaldehyde solution at pH 7.0 for 24 h at 4°C. Paraffin embedding, sectioning, and staining with hematoxylin and eosin (HE) were performed as previously described [7, 8]. The HE sections were photographed under a light microscope (DP-71, Olympus, Tokyo, Japan). The histological findings were compared to the corresponding findings from SD-OCT images.

### 2.5. Electron Microscopy

Electron microscopy was performed using eyes enucleated from S334ter rats on P22, P36, and P61 and from SD rats on P69 according to a previously described method [7, 8]. The eye contralateral to that used for histological examination was examined under an electron microscopy. Immediately after enucleation, the eyes were fixed with 2.5% glutaraldehyde and 2% paraformaldehyde solution at pH 7.4 for 24 h at 4°C. Similarly to the histological processing, an aliquot of the same fixation solution that was used for the histological study was injected into the anterior chamber. The retina and choroid were dissected out, postfixed in phosphate buffered 1% osmium tetroxide at pH 7.4 for 3 h at 4°C, dehydrated in an ascending series of ethanol series (50%-100%), and embedded in epoxy resin. Thin sections (80-90 nm) were stained in uranyl and lead salt solutions. The sections were photographed by a transmission electron microscope (H-7600, Hitachi, Tokyo, Japan) at 100 kV.

### 2.6. Electroretinography (ERG)

ERG was performed using a Micron® Ganzfeld ERG system (Phoenix Research Labs) according to the manufacturer's instructions, with some modifications. In brief, the light stimulus was fixed at 3.0 cd.s/m^2^, after the stimulus-dependent manner in responses was confirmed by changing the stimulation from 3.0 to 30.0 cd.s/m^2^ in a group of control rats according to the ICEV standard [18]. ERG was recorded at 5 time points starting from P17 to P110 for S334ter rats and at 6 time points from P19 to P112 for SD rats. The rats were dark-adapted for at least 24 h and then anesthetized by the same method for the SD-OCT examination. A reference electrode was placed in the center of the scalp, and a ground electrode was placed in the proximal portion of the tail skin. During the measurement, the body temperature was maintained at 37°C, using a body warmer. The pupils were dilated by eye drops containing a mixture of 0.5% tropicamide and 0.5% phenylephrine hydrochloride. After the corneal surface was anesthetized using 0.4% oxybuprocaine hydrochloride eye drops, a contact-lens electrode (Micron® Ganzfeld ERG; Phoenix Research Labs) was applied directly to the corneal surface. We defined the negative wave just after light stimulation as the a-wave and the most positive peak of the oscillatory potentials as the b-wave. The averages of twenty responses (stimulus interval = 10 s) of both a- and b-waves were used for further analyses. Similarly to the SD-OCT segmentation analysis, we evaluated three or four rats and used the average value of both eyes of the same rat as one observation. The number of rats used in each measurement is described in Table 2 and Figure 5.

### 2.7. Statistical Analyses

All of the statistical analyses were performed using the SPSS software program (version 25, Statistical Package for the Social Sciences, Chicago, IL, USA). The segmentation data from the two groups were compared using a two-way repeated analysis of variance (two-way repeated ANOVA) after the normality and equality of each distribution were confirmed by the Shapiro-Wilk test and Levene's test, respectively. Similar* post hoc* analyses using Tukey's* t*-test were performed between similar age-groups (S334ter vs. SD: P19 vs. P19, P28 vs. P26, P34 vs. P33, P46 vs. P54, P87 vs. P82, and P110 vs. P134, respectively) for SD-OCT segmentation. Similar* post hoc* analyses using Turkey's* t*-test were performed to compare the a- and b-wave amplitudes and the implicit times of a-waves between the two groups (three pairs of age-matched rat groups; S334ter vs SD: P18 vs P19, P21 vs P22, and P110 vs P112, respectively).* P* values < 0.05 were considered to indicate statistical significance.

## 3. Results

### 3.1. The SD-OCT Findings in SD Rats

The typical SD-OCT findings in SD rats are shown in Figures 1(a), 1(c), 1(e), 1(g), and 1(i). The retinal sublayers A−D and the zones equivalent to the photoreceptor inner segment ellipsoid zone (EZ) and the interdigitation zone (IZ) in human SD-OCT [18] were clearly identified in these pictures (S 1, 2, 3). These basic structures consistently appeared in the SD-OCT images from SD rats from P19 to P134.

### 3.2. The Qualitative Analyses of the SD-OCT Findings in relation to the Photoreceptor Structure in the Rhodopsin S334ter Transgenic Rats (Line 4)

**Figure 2:**
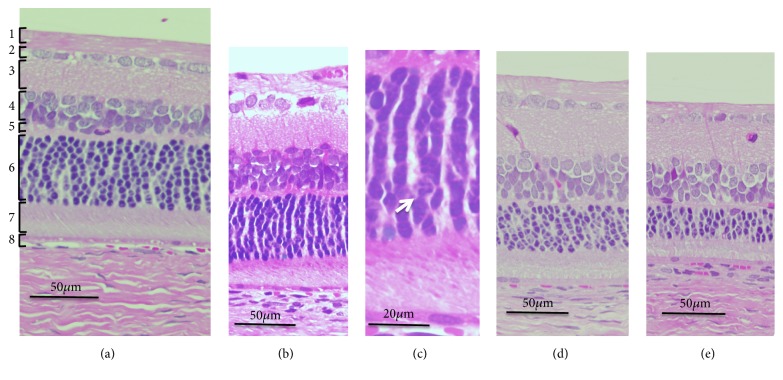
The histological findings of the SD and S334ter rats (hematoxylin and eosin staining). (a) An SD rat on P69. (b) An S334ter rat on P22. (c) The same rat as panel B (high magnification). Arrow indicates an area of reduced chromatin density. (d) The S334ter rat on P36. The S334ter rat on P61. The numeral indicates each retinal layer: 1, nerve fiber layer; 2, ganglion cell layer; 3, inner plexiform layer; 4, inner nuclear layer; 5, outer plexiform layer; 6, outer nuclear layer; 7, photoreceptor rod and cone layer; 8, retinal pigment epithelium and choroid.

**Figure 3:**
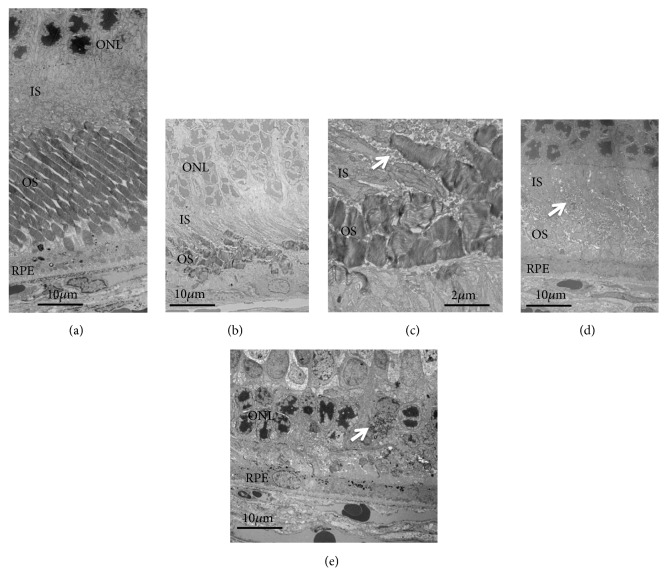
The electron microscopic findings of SD and S334ter rats. (a) An SD rat on P69. (b) An S334ter rat on P22. (c) The same rat as panel (b) (high magnification). Arrows in (c) and (d) indicate extracellular granules. (d) The S334ter rat on P36. (e) The S334ter ray on P61. The arrow in (e) indicates an area of intracellular granule accumulation. Abbreviations, ONL, outer nuclear layer; IS, inner segment; OS, outer segment; RPE, retinal pigment epithelium.

We analyzed the SD-OCT images of the S334ter rats (line 4) to qualitatively characterize the SD-OCT findings in S334ter rats. Typical SD-OCT findings obtained from P19 to P110 are shown in Figures 1(b), 1(d), 1(f), 1(h), and 1(j). In S334ter rats, the retinal sublayer C corresponding to the photoreceptor inner and outer segments became diffusely hyperreflective and did not show a distinctive EZ or IZ, even on P19 (Figure 1(b), S 4). This tendency was consistent from at least P19 to P110 (Figures 1(b), 1(d), 1(f), 1(h), and 1(j); S 4, 5). In addition, the retinal sublayer B, namely, the outer nuclear layer, became progressively thinner and was recognized as a dark linear zone on the SD-OCT images obtained on P87 and P110 (Figures 1(h) and 1(j); S 4, 5). Conversely, the retinal sublayer A (inner layer) appeared to be consistent throughout the observation periods. On comparing these findings to the histological findings obtained from the S334ter rats on P22, P36, and P61 and from SD rats on P69 (Figure 2), despite the regularly arranged appearance of the photoreceptor inner and outer segments observed in the SD rat (Figure 2(a)), over time, their regularity was gradually lost in the S334ter rats, and the photoreceptor layer became progressively degenerated (Figures 2(b), 2(c), 2(d), and 2(e)). Moreover, there were some areas in which the chromatin density became weak and in which aggregation was observed in the outer nuclear layer, suggesting the gradual progression of cell death mechanisms (Figure 2(c)). Electron microscopy revealed that although the photoreceptor inner and outer segments appeared regularly arranged in the SD rat (Figure 3(a)), the structure of the outer segment was severely deteriorated, even on P22, and that the photoreceptor layer became thin (Figures 3(b) and 3(c)). In Figures 3(b) and 3(c), the length of the outer segment became extremely short in comparison to that in the SD rats (Figure 3(a)) and each outer segment was disoriented, despite the fact that the discs were relatively well-packed. In addition, there were numerous granule-like materials in the interphotoreceptor matrix (extracellular vesicles, Figure 3(c), arrow). This tendency was also observed in the image from P36 (Figure 3(d), arrow). Furthermore, the basic structure of the inner and outer segments almost disappeared until P61, and granular materials with a high electron density were observed in the photoreceptor cells (Figure 3(e), arrow). These degenerative changes that occurred in the photoreceptor inner and outer segment layer seemed to contribute to the diffuse hyperreflective changes and the loss of EZ and IZ seen in the SD-OCT images of the S334ter (line 4) rats.

### 3.3. The Quantitative Analyses of the SD-OCT Findings from the S334ter Transgenic Rats (Line 4)

**Figure 4:**
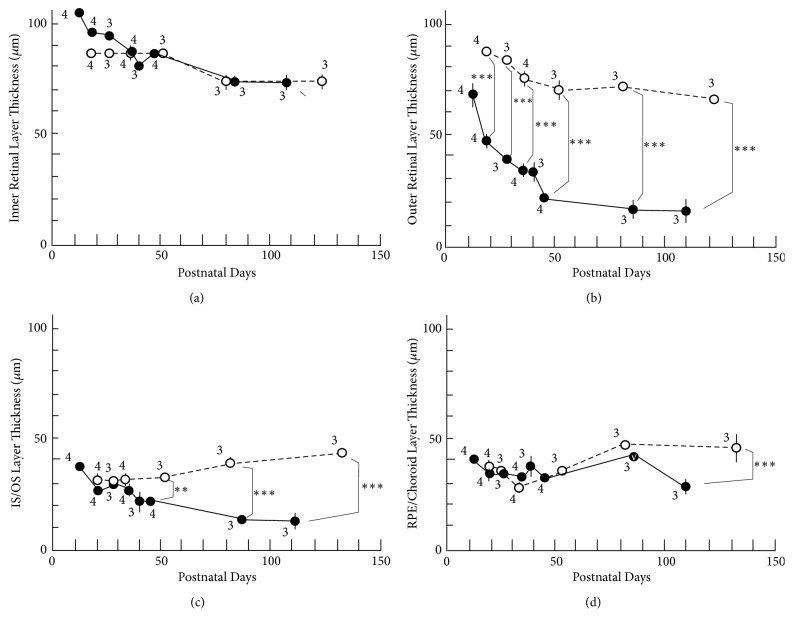
The longitudinal changes in the thicknesses of retinal sublayers. Open circles, SD rats; closed circles, S334ter rats. (a) Thickness changes in the inner retinal sublayer. (b) The thickness of the outer retinal sublayer. (c) The thickness of the photoreceptor inner and outer (IS/OS) sublayer. (d) The thickness of the combined RPE and choroid sublayer. Statistical significance: *∗∗*,* P* < 0.01; *∗∗∗*,* P *< 0.001 (Turkey's* t*-test). Bars indicate standard error. The numeral alongside each circle indicates the number of rats used for each measurement.

The longitudinal changes in the thickness of the retinal sublayer are shown in Table 1 and Figure 4. There was no statistically significant difference in the thickness of the sublayer A between the S334ter and SD rats at any point (Figure 4(a)). However, there were statistically significant differences in the thickness of sublayer B between the two groups throughout the observation periods from P19 (Figure 4(b)). On comparing this finding to the SD-OCT images, the rapid decrease in the thickness of the outer nuclear layer contributed to the phenomenon (Figure 2). In sublayer C, the thickness of the photoreceptor inner and outer segments of the S334ter rats became significantly thinner in comparison to the SD rats after P45 (Figure 4(c)). This tendency corresponded to what we observed in the historical and ultrastructural findings (Figures 2 and 3). In addition, a statistically significant difference was observed in the thickness of sublayer D of the S334ter rats only on P110 (Figure 4(d)). After P110, when the photoreceptor inner and outer segments had already become severely degenerated in the S334ter rats, the thickness of the combined RPE and choroid became significantly thinner in comparison to the SD rats.

### 3.4. The Relationship between the SD-OCT Findings and the ERG Findings in the S334ter Transgenic Rats (Line 4)

**Figure 5:**
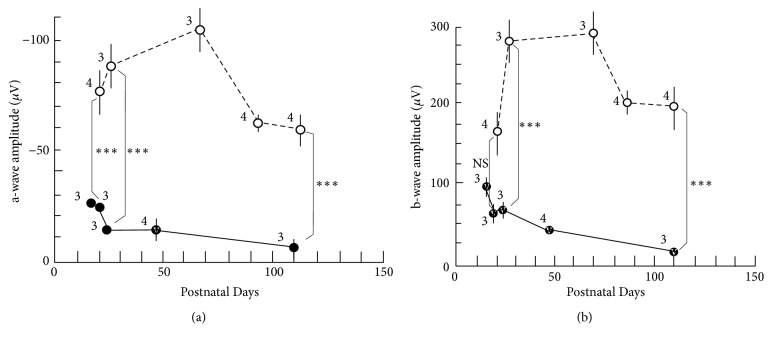
Longitudinal changes in the amplitudes of the a- and b-waves on ERG. Open circles, SD rats; closed circles, S334ter rats. (a) The longitudinal changes in the a-wave. (b) The longitudinal changes in the b-wave. Statistical significance: NS, not significant, *∗∗∗*,* P *< 0.001 (Turkey's* t*-test). Bars indicate standard error. The numeral alongside each circle indicates the number of rats used for each measurement.

**Figure 6:**
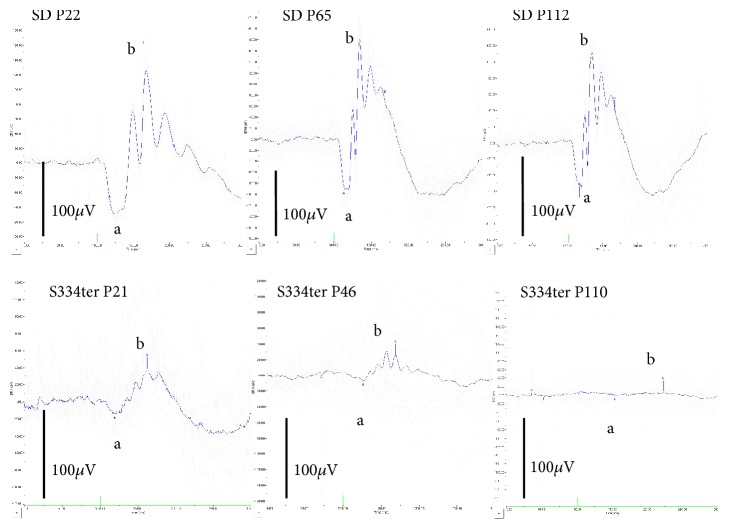
Representative ERG waveforms of the SD and S334ter rats. The upper panels show ERG waveforms of SD rats on P22, P65, and P112, respectively. The lower panels show ERG waveform of S334ter rats on P21, P46, and P110, respectively. Abbreviations: a, a-wave; b, b-wave.

The longitudinal changes in the amplitudes of both the a- and b-waves on ERG are presented in Table 2 and Figure 5. In the a-wave of S334ter rats, the amplitude was significantly reduced in comparison to SD rats at all points after P19 (Figure 5(a)). Similarly, the amplitude of the b-wave in S334ter rats was significantly decreased after P22; however, the difference on P19 was not statistically significant (Figure 5(b)). The deterioration in the a-wave appeared to start earlier than the rapid reduction in the thickness of the sublayer B (Figures 4(b) and 5(a)). On the contrary, the implicit times of a-waves were relatively well maintained up to P46 (Table 2). There was no statistical significance in the implicit times of a-waves between S334ter and SD rats at P18 vs P19 (*P* = 1.000), P21 vs P22 (*P* = 1.000), and P46 vs P65 (*P* = 0.993), respectively, although there was significant difference between S334ter and SD rats at P110 vs P112 (*P* < 0.001, Turkey* t*-test). Representative ERG findings are presented in Figure 6. While the waveforms of the SD rats were consistent throughout the observation periods, those in the S334ter rats progressively deteriorated and finally disappeared on P110 (Figure 6; S 6, 7).

## 4. Discussion

In the present study, we characterized the SD-OCT findings observed during the processes of retinal degeneration in rhodopsin S334ter transgenic rats (line 4) in relation to the histological, ultrastructural, and ERG features. Although the SD-OCT findings related to retinal degeneration in rhodopsin P23H transgenic rats (lines 1 and 2) were previously reported [8, 10, 11], there have been no reports on retinal degeneration in S334ter rats except for the TD-OCT findings of S334ter rats (line 3) [16]. Because the molecular mechanisms of the photoreceptor cell death are reportedly different between the P23H (Class 2) and the S334ter (Class 1) rhodopsin mutations [19, 20], it is interesting to determine whether there are any differences in the SD-OCT findings and their relationships with morphological and electrophysiological features during the processes of retinal degeneration between these two mutations. In the Class 2 rhodopsin mutations, because the mutations are located in the N-terminal (intradiscal) portion, proper molecular folding is impaired during translation and, consequently, unfolded rhodopsin molecules accumulated in the endoplasmic reticulum (ER), resulting in severe ER stress [21]. In contrast, in Class 1 mutations, because of the defective trafficking signals in the C-terminal portion, rhodopsin mutants show defective integration into the disc membrane, which resulted in the accumulation of mislocalized rhodopsin mutants in the inner segment, outer nuclear layer, and synaptic terminus, findings that were reported from studies using several models of Class 1 mutants [5, 22, 23]. Although the accumulated rhodopsin mutant molecules are excreted into the interphotoreceptor matrix as extracellular vesicles, retinal photoreceptor cell death rapidly occurs [5, 23, 24]. In addition, because photoreceptor degeneration proceeded, even under without light stimulation, the intra- and extracellular accumulation of the rhodopsin mutants may play roles in cell death [24].

In a previous study using P23H rats, SD-OCT was sensitive enough to detect even slight disarrangement of the disc structures in the outer segments [8]. Because photoreceptor degeneration progresses much faster and more severely in S334ter rats than in P23H rats, we concluded that the changes occurred in the photoreceptor layer of S334ter rats; i.e., the disoriented and short outer segments and aggregated extracellular vesicles were detected on SD-OCT images as a diffuse hyperreflective zone without any obvious EZ or IZ structures (Figure 1). However, contrary to our expectations, because the abnormal SD-OCT findings in the photoreceptor layer were common between the P23H and the S334ter rats, we could not differentiate between the retinal degeneration caused by P23H or S334ter based on SD-OCT images alone. In addition, because we previously reported that the SD-OCT images of retinal degeneration in RCS rats were also characterized by a diffuse hyperreflective zone in the inner and outer segment layer [7], we concluded that the degenerative changes in the inner and/or outer segments were associated with a loss of optical uniformity and, consequently, caused scattering and hyperreflection which were detected by SD-OCT; these SD-OCT findings may be observed with various causative gene mutations.

In ERG, the amplitudes of both the a- and b-waves were severely deteriorated in the S334ter rats (Figure 6). This severity did not correspond with the SD-OCT findings, because the thickness of the sublayer B in the S334ter rats was reduced by an average of 51.903% in comparison to SD rats on P20 while there was no significant difference in the thicknesses of the sublayer C (Figure 4). In contrast, the amplitudes of the a-wave in the S334ter rats on P19 had already decreased by an average of 35.566% in comparison to that observed in the SD rats (Figure 5). This result in the S334ter rats was different from what we previously observed in P23H rats (line 2), in which the thickness of the sublayer B was correlated with the amplitudes of both the a- and b-waves [8]. These observations suggest that the functional damage of the photoreceptors in the S334ter rats is more severe than what can be estimated from measuring the thickness of the retinal sublayers and that the pathological and qualitative damages in the photoreceptors should be considered. As for the implicit times of a-waves, they were relatively maintained in the S334ter rats until P46 and they became deteriorated toward P110. Previously, we found similar longitudinal changes of ERG in the P23H (line 2) rats [8]. It is possible that the relative preservation of the implicit times of a-waves is one of characteristics of the retinal degeneration associated with mutations in the rhodopsin genes.

In conclusion, the SD-OCT assessments of S334ter transgenic rats (line 4) demonstrate the qualitative abnormalities in photoreceptor degeneration as the diffuse hyperreflective changes in the inner and outer segment layer and the loss of the EZ and IZ; these changes indicate the deterioration of the inner and outer segments and the accumulated extracellular vesicles. In addition, the OCT examinations provide quantitative information that demonstrates the progressive thinning of the outer nuclear layer, the inner and outer segment layer, and the RPE and choroid layer. The functional aspect should be estimated by the combination of both quantitative and qualitative changes in the retinal components. The results obtained in the present study can be applied in the clinical field when the pathologic stage needs to be estimated in patients with RP associated with mutations in the rhodopsin gene.

## Figures and Tables

**Table 1:** Retinal layer thickness (*µ*m) of SD and S334ter rats.

SD Rat					
Age	No of Rats	Inner Retinal Layer (A)	Outer Retinal Layer (B)	IS/OS Layer (C)	RPE + Choroid (D)
P19	4	87.864 ± 2.200	87.462 ± 1.079	31.687 ± 1.691	37.170 ± 2.625
P26	3	87.777 ± 1.550	84.206 ± 0.158	32.227 ± 0.038	35.853 ± 0.074
P33	4	87.818 ± 3.323	77.045 ± 2.449	28.726 ± 2.232	29.046 ± 2.568
P54	3	87.321 ± 2.092	71.314 ± 4.470	35.450 ± 1.538	35.842 ± 1.799
P82	3	73.090 ± 2.367	73.397 ± 0.865	41.552 ± 1.465	51.048 ± 0.665
P134	3	72.677 ± 2.416	69.898± 0.158	43.600 ± 0.333	52.398 ± 5.925

S334ter Rat					
Age	No of Rats	Inner Retinal Layer (A)	Outer Retinal Layer (B)	IS/OS Layer (C)	RPE + Choroid (D)

P13	4	104.730 ± 1.663	69.715 ± 4.082	37.180 ± 1.065	40.423 ± 1.065
P20	4	94.303 ± 1.675	45.395 ± 1.836	28.615 ± 0.981	34.283 ± 1.723
P28	3	93.769 ± 0.657	39.072 ± 1.679	31.390 ± 0.813	35.484 ± 0.861
P34	4	89.317 ± 2.436	35.749 ± 2.347	28.918 ± 1.543	32.845 ± 1.323
P40	3	79.263 ± 4.287	35.038 ± 2.851	24.559 ± 4.087	39.668 ± 4.320
P46	4	85.459 ± 1.976	23.127 ± 1.369	24.823 ± 0.884	33.632 ± 1.467
P87	3	76.106 ± 0.544	19.050 ± 3.660	16.481 ± 0.989	46.0614 ± 1.429
P110	3	76.0799 ± 3.076	17.059 ± 4.778	16.881 ± 3.266	29.467 ± 4.272

Values indicate mean ± standard error.

No, number.

**Table 2:** ERG amplitudes (*µ*V) of SD and S334ter rats.

SD Rats			
Age	No of Rats	a-wave	b-wave
P19	4	-79.983 ± 9.861	168.425 ± 27.975
P22	3	-89.848 ± 9.776	281.515 ± 29.500
P65	3	-106.062 ± 9.840	298.195 ± 24.070
P92	4	-63.557 ± 3.953	202.557 ± 14.166
P112	4	-61.691 ± 6.861	199.299 ± 23.208

S334ter Rats			
Age	No of Rats	a-wave	b-wave

P17	3	-25.566 ± 2.732	97.607 ± 12.585
P18	3	-24.618 ± 0.845	62.379 ± 11.422
P21	3	-12.202 ± 0.049	65.274 ± 11.117
P46	4	-13.750 ± 2.394	40.661 ± 2.872
P110	3	-5.542 ± 1.254	14.684 ± 3.789

Values indicate mean ± standard error.

No, number.

## Data Availability

The data used to support the findings of this study are available from the corresponding author upon request.

## Supplementary Materials

Supplementary Materials Supplementary image 1 (S 1): definition of retinal sublayers A, B, C, and D, ELM, EZ, and IZ, and comparison between a representative SD-OCT image and histological findings. Abbreviations: ELM, external limiting membrane; EZ, inner segment ellipsoid zone; IZ, interdigitation zone. Supplementary image 2 (S 2): representative OCT images of four eyes of SD rats at P19, P26, and P33, respectively. Bars indicate 100*µ*m. Supplementary image 3 (S 3): representative OCT images of four eyes of SD rats at P54, P82, and P134, respectively. Bars indicate 100*µ*m. Supplementary image 4 (S 4): representative OCT images of four eyes of S334ter transgenic rats at P13, P20, P28, and P34, respectively. Bars indicate 100*µ*m. Supplementary image 5 (S 5): representative OCT images of four eyes of S334ter transgenic rats at P40, P46, P87, and P110, respectively. Bars indicate 100*µ*m. Supplementary image 6 (S 6): representative ERG waves of three eyes of SD rats at P19, P22, P65, P92, and P112, respectively. Arrows indicate the time point of light stimulation. Bars indicate 100ms. The y-axis shows amplitude in *µ*V. Supplementary image 7 (S 7): representative ERG waves of three eyes of S334ter transgenic rats at P17, P18, P21, P46, and P110, respectively. Arrows indicate the time point of light stimulation. Bars indicate 100ms. The y-axis shows amplitude in *µ*V.
