# Supplementary figures and images for: The Spectral-Domain Optical Coherence Tomography Findings Associated with the Morphological and Electrophysiological Changes in a Rat Model of Retinal Degeneration, Rhodopsin S334ter-4 Rats
Source: Biomed Res Int. 2018 Nov 15;2018:5174986. doi: 10.1155/2018/5174986 (PMC6276524; doi:10.1155/2018/5174986)

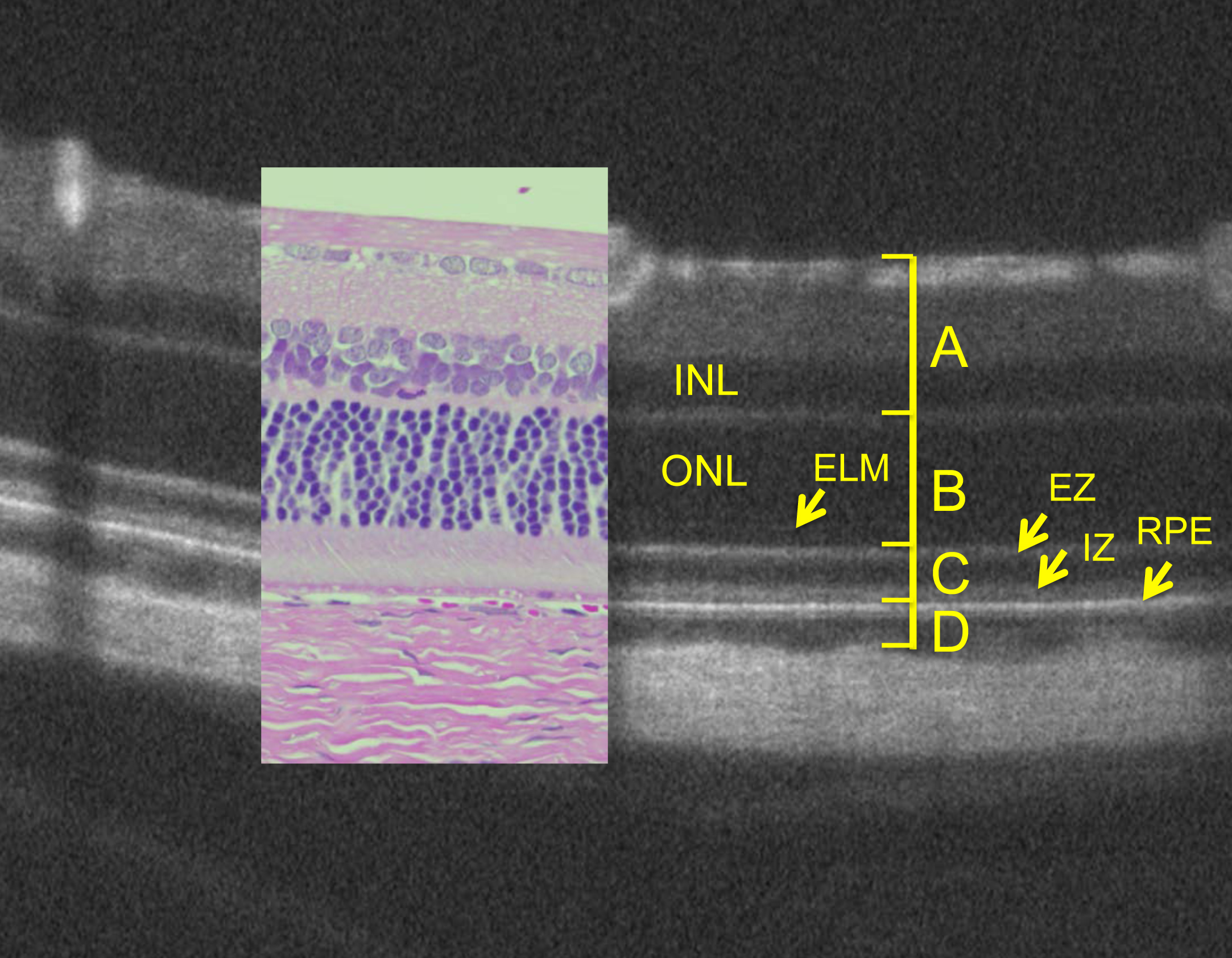

Supplement: Supplementary Materials — Supplementary image 1 (S 1): definition of retinal sublayers A, B, C, and D, ELM, EZ, and IZ, and comparison between a representative SD-OCT image and histological findings. Abbreviations: ELM, external limiting membrane; EZ, inner segment ellipsoid zone; IZ, interdigitation zone. Supplementary image 2 (S 2): representative OCT images of four eyes of SD rats at P19, P26, and P33, respectively. Bars indicate 100µm. Supplementary image 3 (S 3): representative OCT images of four eyes of SD rats at P54, P82, and P134, respectively. Bars indicate 100µm. Supplementary image 4 (S 4): representative OCT images of four eyes of S334ter transgenic rats at P13, P20, P28, and P34, respectively. Bars indicate 100µm. Supplementary image 5 (S 5): representative OCT images of four eyes of S334ter transgenic rats at P40, P46, P87, and P110, respectively. Bars indicate 100µm. Supplementary image 6 (S 6): representative ERG waves of three eyes of SD rats at P19, P22, P65, P92, and P112, respectively. Arrows indicate the time point of light stimulation. Bars indicate 100ms. The y-axis shows amplitude in µV. Supplementary image 7 (S 7): representative ERG waves of three eyes of S334ter transgenic rats at P17, P18, P21, P46, and P110, respectively. Arrows indicate the time point of light stimulation. Bars indicate 100ms. The y-axis shows amplitude in µV. [file 5174986.f1.zip › S 1.jpg]

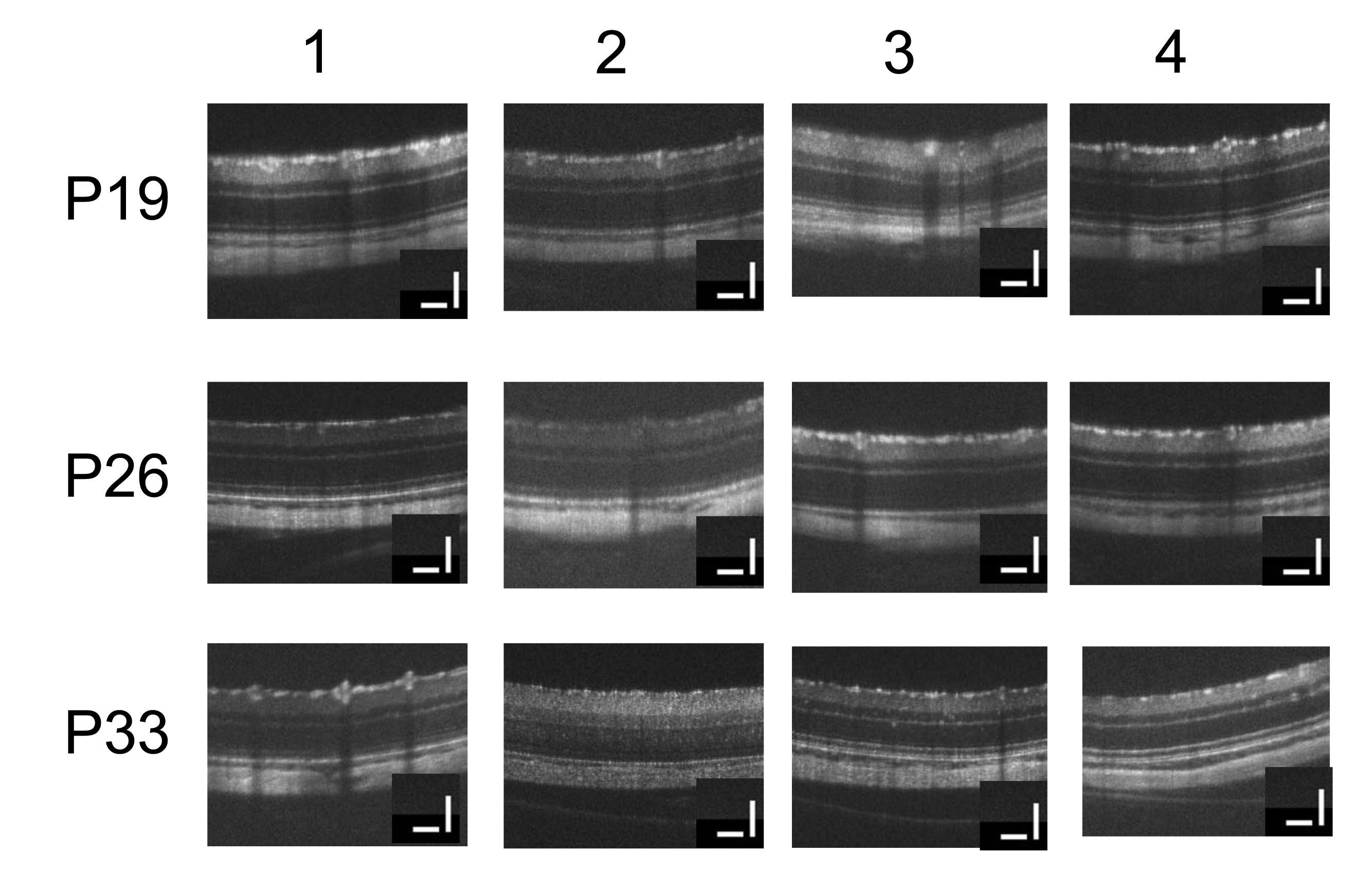

Supplement: Supplementary Materials — Supplementary image 1 (S 1): definition of retinal sublayers A, B, C, and D, ELM, EZ, and IZ, and comparison between a representative SD-OCT image and histological findings. Abbreviations: ELM, external limiting membrane; EZ, inner segment ellipsoid zone; IZ, interdigitation zone. Supplementary image 2 (S 2): representative OCT images of four eyes of SD rats at P19, P26, and P33, respectively. Bars indicate 100µm. Supplementary image 3 (S 3): representative OCT images of four eyes of SD rats at P54, P82, and P134, respectively. Bars indicate 100µm. Supplementary image 4 (S 4): representative OCT images of four eyes of S334ter transgenic rats at P13, P20, P28, and P34, respectively. Bars indicate 100µm. Supplementary image 5 (S 5): representative OCT images of four eyes of S334ter transgenic rats at P40, P46, P87, and P110, respectively. Bars indicate 100µm. Supplementary image 6 (S 6): representative ERG waves of three eyes of SD rats at P19, P22, P65, P92, and P112, respectively. Arrows indicate the time point of light stimulation. Bars indicate 100ms. The y-axis shows amplitude in µV. Supplementary image 7 (S 7): representative ERG waves of three eyes of S334ter transgenic rats at P17, P18, P21, P46, and P110, respectively. Arrows indicate the time point of light stimulation. Bars indicate 100ms. The y-axis shows amplitude in µV. [file 5174986.f1.zip › S 2. OCT-SD1. jpeg]

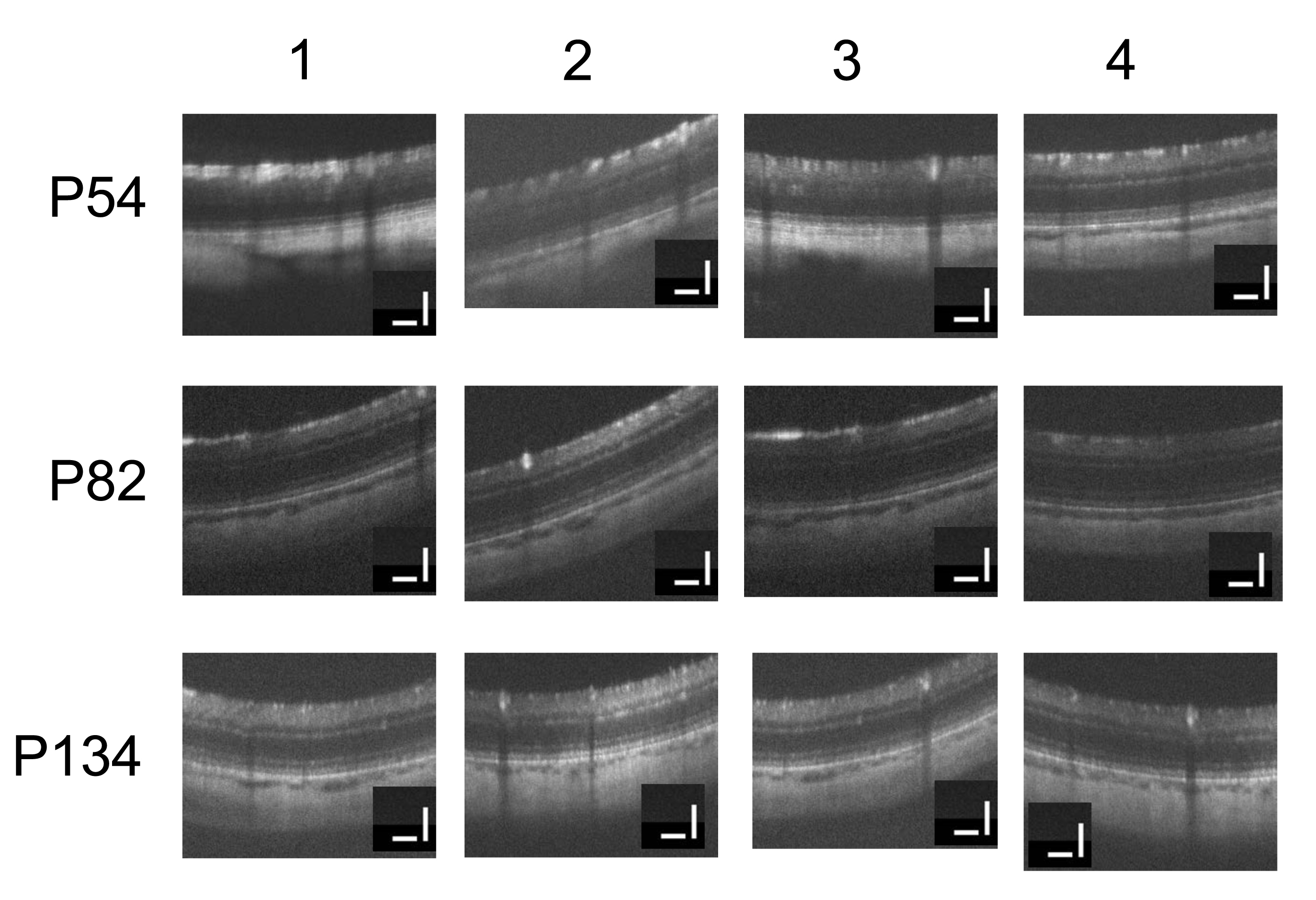

Supplement: Supplementary Materials — Supplementary image 1 (S 1): definition of retinal sublayers A, B, C, and D, ELM, EZ, and IZ, and comparison between a representative SD-OCT image and histological findings. Abbreviations: ELM, external limiting membrane; EZ, inner segment ellipsoid zone; IZ, interdigitation zone. Supplementary image 2 (S 2): representative OCT images of four eyes of SD rats at P19, P26, and P33, respectively. Bars indicate 100µm. Supplementary image 3 (S 3): representative OCT images of four eyes of SD rats at P54, P82, and P134, respectively. Bars indicate 100µm. Supplementary image 4 (S 4): representative OCT images of four eyes of S334ter transgenic rats at P13, P20, P28, and P34, respectively. Bars indicate 100µm. Supplementary image 5 (S 5): representative OCT images of four eyes of S334ter transgenic rats at P40, P46, P87, and P110, respectively. Bars indicate 100µm. Supplementary image 6 (S 6): representative ERG waves of three eyes of SD rats at P19, P22, P65, P92, and P112, respectively. Arrows indicate the time point of light stimulation. Bars indicate 100ms. The y-axis shows amplitude in µV. Supplementary image 7 (S 7): representative ERG waves of three eyes of S334ter transgenic rats at P17, P18, P21, P46, and P110, respectively. Arrows indicate the time point of light stimulation. Bars indicate 100ms. The y-axis shows amplitude in µV. [file 5174986.f1.zip › S 3. SD-OCT2]

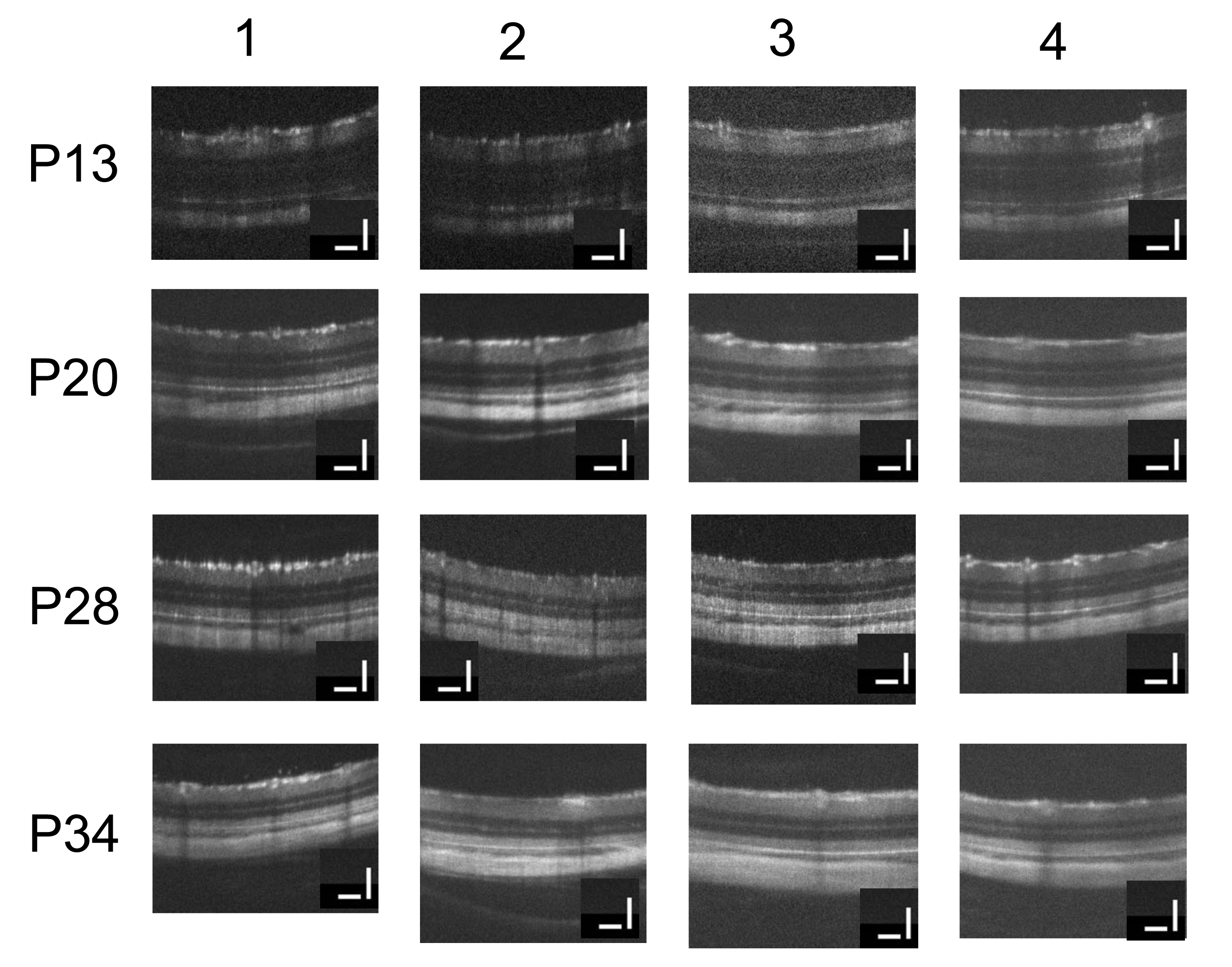

Supplement: Supplementary Materials — Supplementary image 1 (S 1): definition of retinal sublayers A, B, C, and D, ELM, EZ, and IZ, and comparison between a representative SD-OCT image and histological findings. Abbreviations: ELM, external limiting membrane; EZ, inner segment ellipsoid zone; IZ, interdigitation zone. Supplementary image 2 (S 2): representative OCT images of four eyes of SD rats at P19, P26, and P33, respectively. Bars indicate 100µm. Supplementary image 3 (S 3): representative OCT images of four eyes of SD rats at P54, P82, and P134, respectively. Bars indicate 100µm. Supplementary image 4 (S 4): representative OCT images of four eyes of S334ter transgenic rats at P13, P20, P28, and P34, respectively. Bars indicate 100µm. Supplementary image 5 (S 5): representative OCT images of four eyes of S334ter transgenic rats at P40, P46, P87, and P110, respectively. Bars indicate 100µm. Supplementary image 6 (S 6): representative ERG waves of three eyes of SD rats at P19, P22, P65, P92, and P112, respectively. Arrows indicate the time point of light stimulation. Bars indicate 100ms. The y-axis shows amplitude in µV. Supplementary image 7 (S 7): representative ERG waves of three eyes of S334ter transgenic rats at P17, P18, P21, P46, and P110, respectively. Arrows indicate the time point of light stimulation. Bars indicate 100ms. The y-axis shows amplitude in µV. [file 5174986.f1.zip › S 4. OCT-S334ter1]

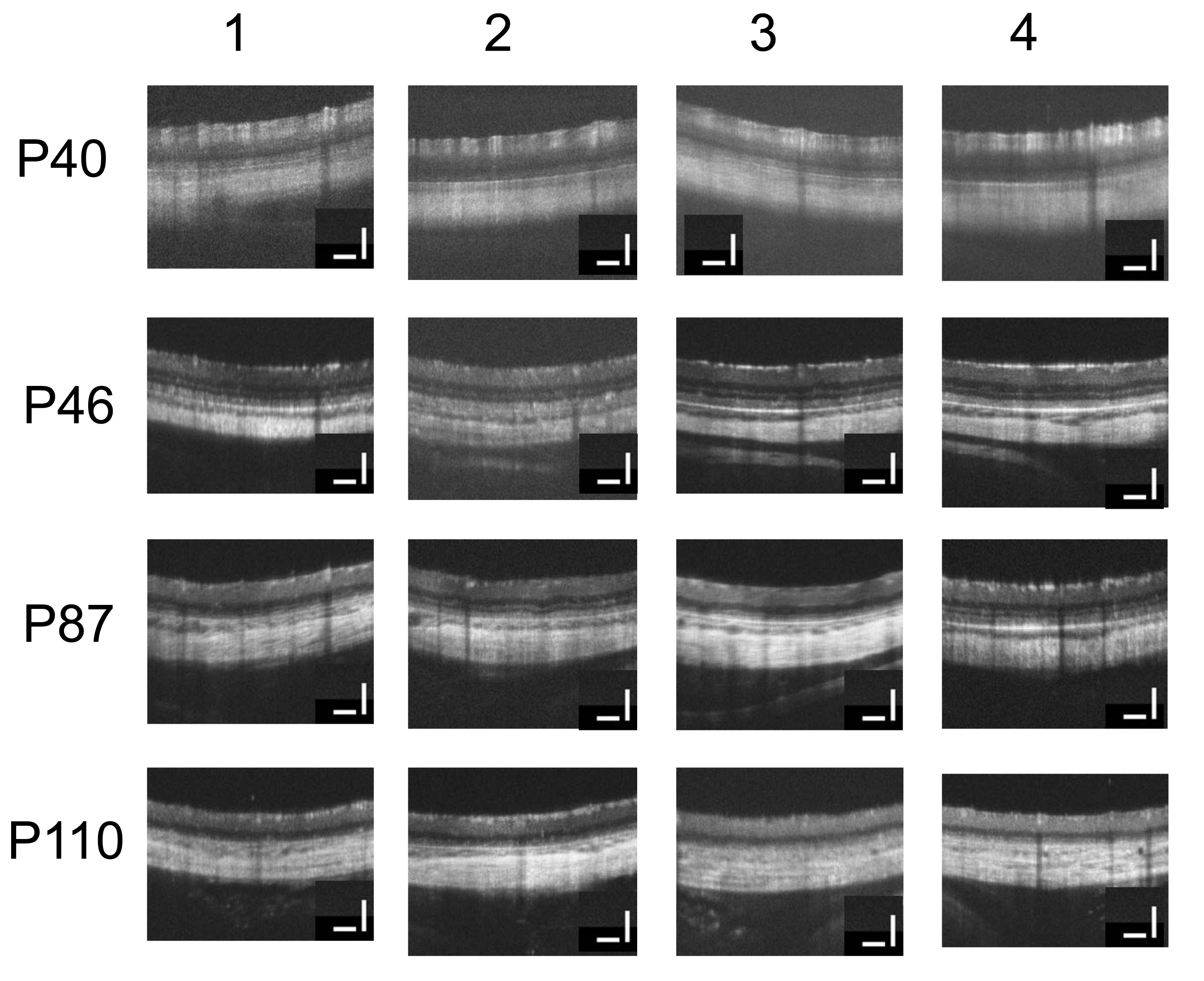

Supplement: Supplementary Materials — Supplementary image 1 (S 1): definition of retinal sublayers A, B, C, and D, ELM, EZ, and IZ, and comparison between a representative SD-OCT image and histological findings. Abbreviations: ELM, external limiting membrane; EZ, inner segment ellipsoid zone; IZ, interdigitation zone. Supplementary image 2 (S 2): representative OCT images of four eyes of SD rats at P19, P26, and P33, respectively. Bars indicate 100µm. Supplementary image 3 (S 3): representative OCT images of four eyes of SD rats at P54, P82, and P134, respectively. Bars indicate 100µm. Supplementary image 4 (S 4): representative OCT images of four eyes of S334ter transgenic rats at P13, P20, P28, and P34, respectively. Bars indicate 100µm. Supplementary image 5 (S 5): representative OCT images of four eyes of S334ter transgenic rats at P40, P46, P87, and P110, respectively. Bars indicate 100µm. Supplementary image 6 (S 6): representative ERG waves of three eyes of SD rats at P19, P22, P65, P92, and P112, respectively. Arrows indicate the time point of light stimulation. Bars indicate 100ms. The y-axis shows amplitude in µV. Supplementary image 7 (S 7): representative ERG waves of three eyes of S334ter transgenic rats at P17, P18, P21, P46, and P110, respectively. Arrows indicate the time point of light stimulation. Bars indicate 100ms. The y-axis shows amplitude in µV. [file 5174986.f1.zip › S 5. OCT-S334ter2]

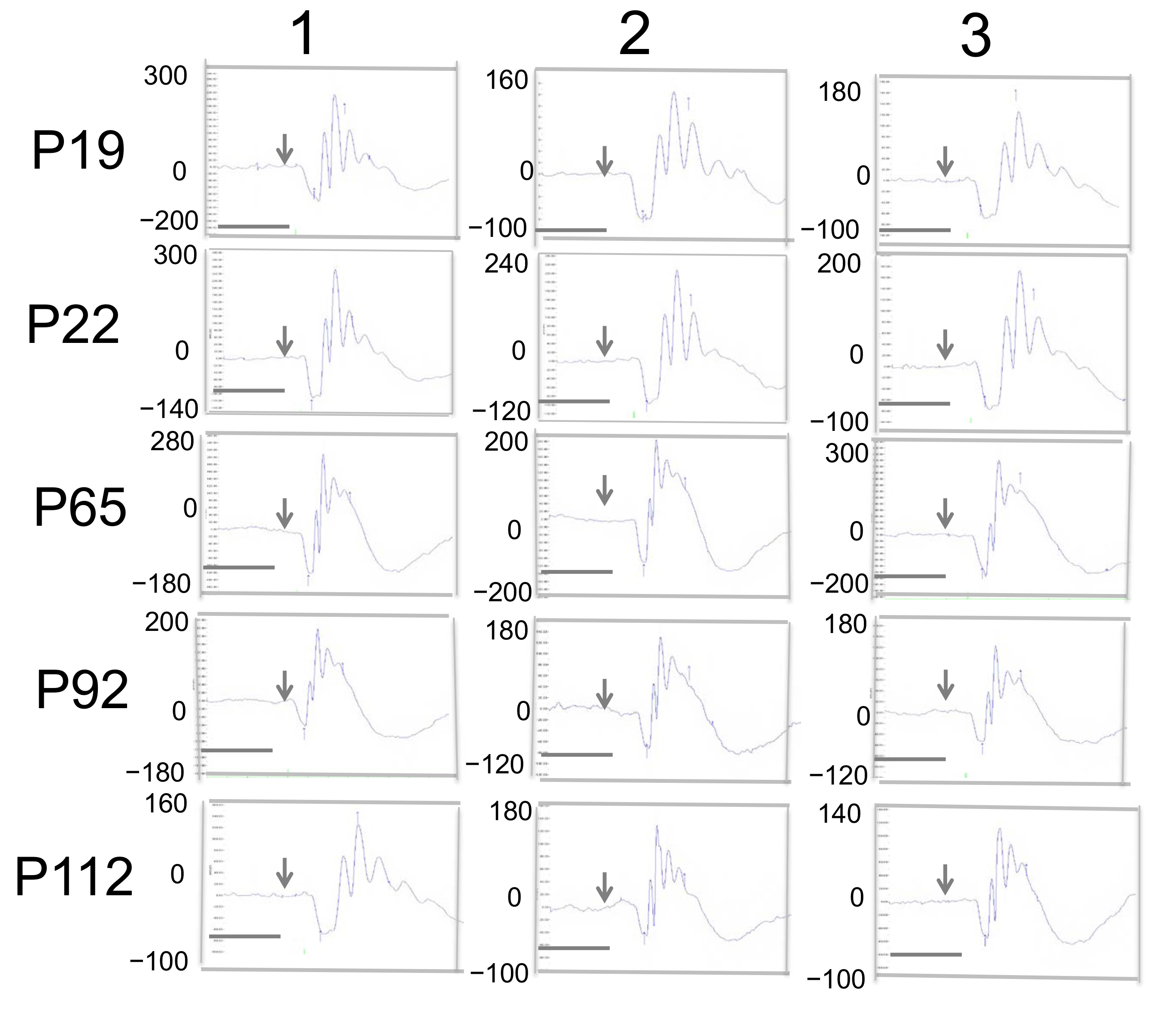

Supplement: Supplementary Materials — Supplementary image 1 (S 1): definition of retinal sublayers A, B, C, and D, ELM, EZ, and IZ, and comparison between a representative SD-OCT image and histological findings. Abbreviations: ELM, external limiting membrane; EZ, inner segment ellipsoid zone; IZ, interdigitation zone. Supplementary image 2 (S 2): representative OCT images of four eyes of SD rats at P19, P26, and P33, respectively. Bars indicate 100µm. Supplementary image 3 (S 3): representative OCT images of four eyes of SD rats at P54, P82, and P134, respectively. Bars indicate 100µm. Supplementary image 4 (S 4): representative OCT images of four eyes of S334ter transgenic rats at P13, P20, P28, and P34, respectively. Bars indicate 100µm. Supplementary image 5 (S 5): representative OCT images of four eyes of S334ter transgenic rats at P40, P46, P87, and P110, respectively. Bars indicate 100µm. Supplementary image 6 (S 6): representative ERG waves of three eyes of SD rats at P19, P22, P65, P92, and P112, respectively. Arrows indicate the time point of light stimulation. Bars indicate 100ms. The y-axis shows amplitude in µV. Supplementary image 7 (S 7): representative ERG waves of three eyes of S334ter transgenic rats at P17, P18, P21, P46, and P110, respectively. Arrows indicate the time point of light stimulation. Bars indicate 100ms. The y-axis shows amplitude in µV. [file 5174986.f1.zip › S 6. ERG SD.jpg]

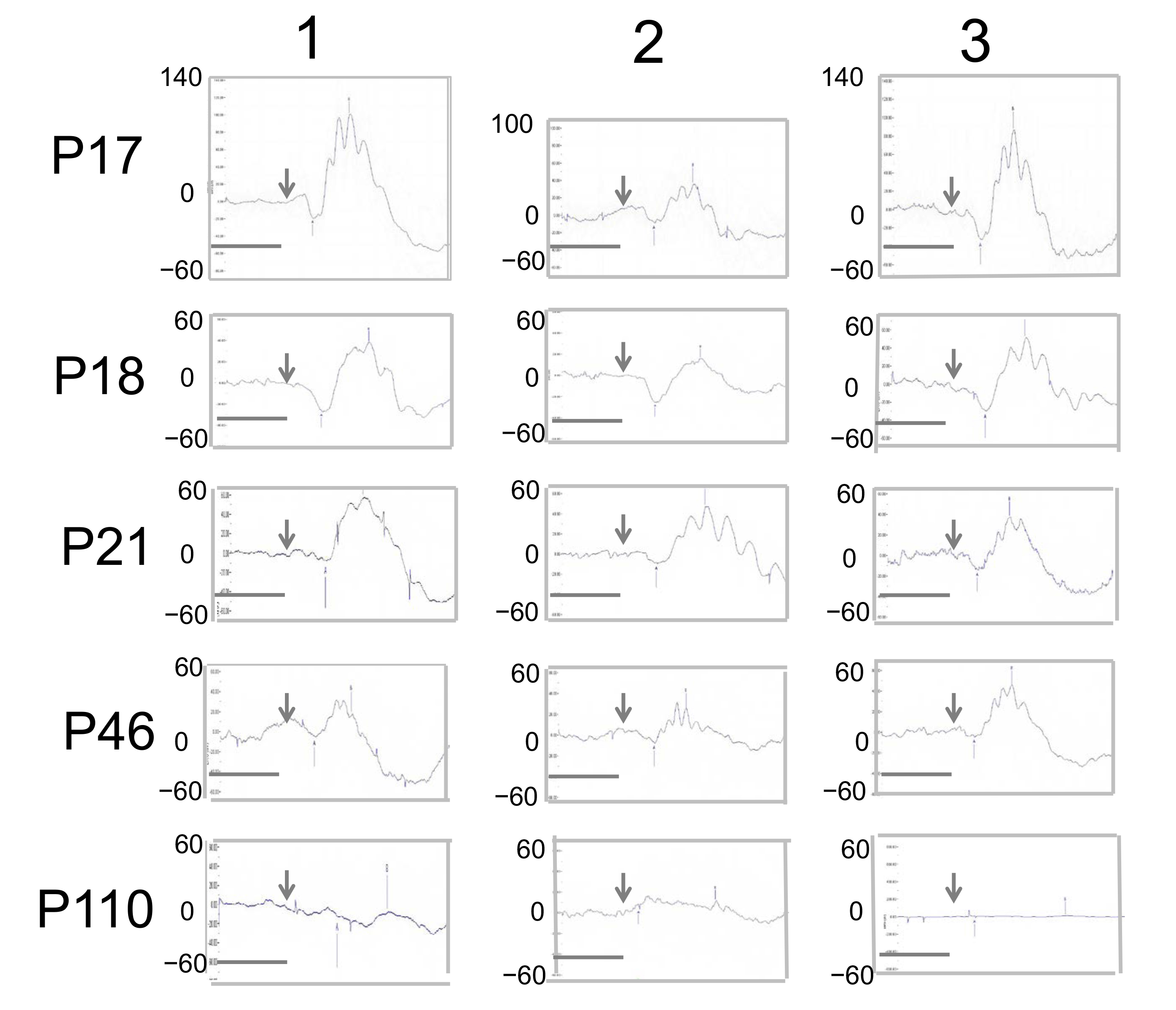

Supplement: Supplementary Materials — Supplementary image 1 (S 1): definition of retinal sublayers A, B, C, and D, ELM, EZ, and IZ, and comparison between a representative SD-OCT image and histological findings. Abbreviations: ELM, external limiting membrane; EZ, inner segment ellipsoid zone; IZ, interdigitation zone. Supplementary image 2 (S 2): representative OCT images of four eyes of SD rats at P19, P26, and P33, respectively. Bars indicate 100µm. Supplementary image 3 (S 3): representative OCT images of four eyes of SD rats at P54, P82, and P134, respectively. Bars indicate 100µm. Supplementary image 4 (S 4): representative OCT images of four eyes of S334ter transgenic rats at P13, P20, P28, and P34, respectively. Bars indicate 100µm. Supplementary image 5 (S 5): representative OCT images of four eyes of S334ter transgenic rats at P40, P46, P87, and P110, respectively. Bars indicate 100µm. Supplementary image 6 (S 6): representative ERG waves of three eyes of SD rats at P19, P22, P65, P92, and P112, respectively. Arrows indicate the time point of light stimulation. Bars indicate 100ms. The y-axis shows amplitude in µV. Supplementary image 7 (S 7): representative ERG waves of three eyes of S334ter transgenic rats at P17, P18, P21, P46, and P110, respectively. Arrows indicate the time point of light stimulation. Bars indicate 100ms. The y-axis shows amplitude in µV. [file 5174986.f1.zip › S 7. ERG S334ter.jpg]
